# Comprehensive Analysis of Mitochondrial Dynamics Alterations in Heart Diseases

**DOI:** 10.3390/ijms24043414

**Published:** 2023-02-08

**Authors:** Giampaolo Morciano, Caterina Boncompagni, Daniela Ramaccini, Gaia Pedriali, Esmaa Bouhamida, Elena Tremoli, Carlotta Giorgi, Paolo Pinton

**Affiliations:** 1 Department of Medical Sciences, University of Ferrara, 44121 Ferrara, Italy; 2 GVM Care & Research, Maria Cecilia Hospital, 48033 Cotignola, Italy

**Keywords:** mitochondria, heart diseases, quality control mechanisms

## Abstract

The most common alterations affecting mitochondria, and associated with cardiac pathological conditions, implicate a long list of defects. They include impairments of the mitochondrial electron transport chain activity, which is a crucial element for energy formation, and that determines the depletion of ATP generation and supply to metabolic switches, enhanced ROS generation, inflammation, as well as the dysregulation of the intracellular calcium homeostasis. All these signatures significantly concur in the impairment of cardiac electrical characteristics, loss of myocyte contractility and cardiomyocyte damage found in cardiac diseases. Mitochondrial dynamics, one of the quality control mechanisms at the basis of mitochondrial fitness, also result in being dysregulated, but the use of this knowledge for translational and therapeutic purposes is still in its infancy. In this review we tried to understand why this is, by summarizing methods, current opinions and molecular details underlying mitochondrial dynamics in cardiac diseases.

## 1. Introduction

Mitochondria were first discovered in 1856 by the physiologist Von Kölliker as sarcosomes while studying human muscle cells; in 1898, they were named mitochondria by Benda from the combination of two Greek words, mitos (thread) and chondros (granule) [1]. Mitochondria possess a double membrane: an outer mitochondrial membrane (OMM) and an inner mitochondrial membrane (IMM), both delimitate the intermembrane space (IMS). Their structures entail phospholipids which play pivotal roles in the overall dynamics and mitochondrial performance; thereby, defects in their compositions can negatively affect the mitochondrial integrity and permeability [2,3]. Additionally, mitochondria are able to interchange their morphology depending on the physiological environment and developmental phase [4]. The accurate functioning of cardiomyocytes needs a steady harmonization of mitochondrial performance, which is intimately linked to a proper balance of cardiac mitochondrial dynamism and quality control (QC) [5]. Although we are witnessing an increased attention among the scientists on the importance of targeting cardiac mitochondria to dissect further potential therapeutic prevention in various cardiovascular diseases (CVDs), a better understanding of the molecular mechanism of mitochondrial dysfunction focusing on mitochondrial dynamics still remains to be fully elaborated. In the present review article, we will mainly provide a comprehensive report on the morphological changes to which mitochondria are subjected in multiple heart pathologies, drawing particular attention to the mitochondrial dynamics to pave the way for further cardioprotective strategies.

## 2. Most Common Mitochondria-Related Alterations Accompanying Heart Diseases

Mitochondria are the primary energy source for regular cellular function and they are fundamental for the proper physiology of the cardiovascular system. Nevertheless, it is highly recognized that beyond their hallmark role as a powerhouse, mitochondria are the main sites of reactive oxygen species (ROS) generation and lipid synthesis, intimately involved in calcium (Ca^2+^) homeostasis and various related signaling pathways [6]. It is not surprising that aberrations in cardiac mitochondrial functioning, structure and number are key factors with a central role in the pathogenesis of multiple chronic human disorders, manifoldly implicated in the development of heart diseases [7]. Therefore, despite the divergence in heart pathophysiology, mitochondrial impairment arises to be a common mechanism that affects cardiac functioning and survival.

The most common alterations in mitochondrial function associated with cardiac conditions implicate further defects in the mitochondrial electron transport chain (ETC) activity, which is a crucial element for energy formation. Then, this is followed by the depletion of ATP generation and supply, reverse metabolic substrate, enhanced ROS generation, inflammation, as well as an aberration in the core myocardial activity of Ca^2+^ homeostasis [8]. These events result in the impairment of cardiac electrical characteristics, loss of myocyte contractility, altered electrical properties and cardiomyocyte damage [9].

Excessive oxidative stress is a common feature in this field, with predominant role in valves disease [10], atherosclerosis progression [11] and coronary artery disease (CAD) [12]. Mitochondria and ROS are closely associated with inflammation. Indeed, mounting pieces of evidence have illustrated the key role of mitochondrial ROS in stimulating the inflammasome NOD-LRR- and pyrin domain-containing protein 3 (NLRP3) and pyroptosis process [13,14]. Mitochondrial dysfunction also results in a dramatic increase in the permeability of the IMM, triggering in turn a profound involvement of ROS formation, mitochondrial bioenergetics impairment and cell death. One of the causes responsible for IMM permeability is the opening of the mitochondrial permeability transition pore (PTP), primarily involved in RI and associated with intracellular Ca^2+^ overload [15,16,17,18] that leads to apoptosis, necrosis and many other types of cell death.

Moreover, it is well recognized that mitochondria metabolize fatty acids to generate 60–90% of energy through oxidative phosphorylation (OXPHOS), required for the myocardium. Scarcely, the remaining 10–40% of energy is formed by using glucose and carbohydrates to provide sufficient energy for cardiac cells [19,20]. However, this balance in cardiac energy metabolism could be gradually shifted in pathological conditions, resulting in an energy crisis, mitigating cardiac activity, and it is believed to contribute to multiple heart disorders [21,22]. Several lines of evidence point out the switch in metabolism as accompanied by modifications in the expression of several transcriptional factors, including the upregulation of hypoxia-inducible factor-1α (HIF-1α) [23], downregulation of peroxisome proliferator-activated receptor α (PPARα) and PPARγ co-activator-1α (PGC-1α). Collectively, these modifications support glycolysis elevation, a reduction in mitochondrial oxidation, and depletion of ATP production [24], and these events have been described in pressure overload cardiac hypertrophy, ischemic heart diseases and in heart failure (HF) [25,26].

Moreover, some other mitochondrial alterations may also occur in the mitochondrial DNA (mtDNA) or nuclear DNA (nDNA) mutations. Likewise, accumulation of mtDNA causes a drastic increase in mitochondrial dysfunction [27]; mtDNA is a central element for mitochondrial activity, as it encodes 37 genes, implicating in those transfer RNAs (tRNAs) and 2 ribosomal RNAs (rRNAs), and genes encode the subunit enzyme complexes in ETC [28]. The interaction between mtDNA and nDNA is inevitable for regular cardiac mitochondrial functioning. However, mutations in mtDNA are more frequent than in the nuclear genome; this may be highlighted by the fact that mtDNA is more exposed to detrimental agents, unprotected by the histones, minimal DNA repair and that the mtDNA mutations are caused mostly by uncontrolled ROS formation which generates oxidative stress [29]. Many CVDs are reported to be associated to DNA mutagenesis [30,31,32,33,34].

## 3. Mitochondrial Dynamics in Physiology

Eukaryotic cells continuously need a large amount of energy to perform their functions, from DNA remodeling, DNA replication, cell division to cardiac contraction and nerve impulse transmission; this is allowed by mitochondria and handled by several mechanisms responsible for their QC. Mitochondria are able to change their morphology, number, size and location inside the cell, thanks to the coordination of a complex system of mitochondrial dynamics, which represents one of the QC mechanisms. As a consequence, the mitochondrial network acquires a dynamic organization, important for combining biological information within the population and assuring a correct cellular energy supply through the balanced processes of fusion and fission [35]. The mitochondrial dynamics homeostasis is fundamental for the physiognomy of the network and for cell fate; furthermore, these morphologic transitions respond to intracellular signals which can be represented also by stressors and thus associated to diseased traits [36,37,38].

When the network becomes fragmented during the fission process, there is an increase in the number of mitochondria, which acquire a smaller and round shape. In physiology, this condition facilitates either the removal of the damaged organelles through mitophagy [39] or the division of the organelles between daughter cells [40]; in contrast, the elongated mitochondria seen in the fused network are fundamental for the maintenance of a healthy mitochondrial status [41]. These two processes, as we will see, need to be in constant balance between them and among the remaining QC mechanisms.

**Figure 1:**
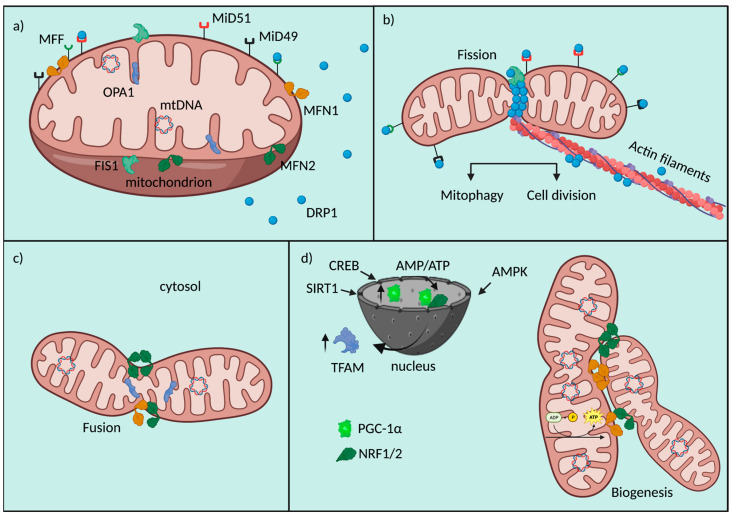
Fusion/Fission machinery. Schematic representation of mitochondrial fusion and fission machinery and their crosstalk with biogenesis. (**a**) Mitochondrial players involved in fusion, fission and biogenesis. (**b**) Mitochondrial fission is orchestrated by a series of molecular events involving OMM receptors (MFF, MiD49, MiD51, FIS1), which help the recruitment of DRP1 to the organelle. DRP1 results to be the main protein effector of mitochondrial fragmentation. (**c**) Mitochondrial fusion is allowed by the tethering properties of MFNs and the function of OPA1 at the IMM. (**d**) Mitochondrial biogenesis is at the crosstalk of a nuclear/mitochondrial gene reprogramming involving the upregulation of PGC-1α, NRF1/2, TFAM, their nuclear interactions and fusion proteins.

Fission consists in the division of both OMM and IMM with the maintenance of the soluble proteins of the IMS and matrix. When fission is needed for mitochondrial division, at early stages, mtDNA is replicated in order to be distributed to daughter mitochondria, and this event is coupled to the formation of ER-mitochondria contacts, predefined sites of mitochondrial fission [42]. The key protein controlling mitochondrial fission in mammals is dynamin-related protein 1 (DRP1), a large GTPase with cytosolic localization. During fission, DRP1 is recruited to the OMM and through oligomerization induces membrane constriction [43]; GTP hydrolysis is necessary for the conformational changes in DRP1 helices and their constrictive functions [44]. DRP1 binds to the receptors’ mitochondrial fission factor (MFF) [45] and mitochondrial dynamics proteins 49 and 51 (MiD49 and MiD51), also called MIEF 1/2 [46], which help its recruitment and function (Figure 1). However, the immediate translocation of DRP1 to fission sites operated by these receptors is not the unique model to explain its involvement in mitochondrial fission. Indeed, Higgs’ group provided evidence for the formation of a supramolecular complex composed by actin filaments and DRP1, supporting mobility inside cells and then fission. Actin assembly would work as a biochemical signal for DRP1 in stimulating its activity [47]. DRP1 protein is structurally composed by four different domains: the N-terminal with GTPase function, a middle part formed by a central stalk necessary for the assembling of DRP1 at the OMM, a variable domain (B-Insert) and the C-terminal with a GTPase effector domain (GED), fundamental for the formation and the stability of higher order structures at the OMM [48,49].

Another essential OMM protein is FIS1, which is involved in the recruitment of DRP1 at the OMM: this is an important regulator of the assembly of mitochondrial fission complexes [50]. In recent years, a novel role for this protein has been discovered in counteracting the fusion process through the inhibition of the GTPase activity of Mitofusins (MFN) 1, MFN2 and optic atrophy factor 1 (OPA1) [51].

On the other hand, fusion consists in the tethering of two neighboring mitochondria that secondly fuse their OMM and IMM. This process inevitably involves the exchange of biological material of different natures, for example, mtDNA [52]. Usually, material exchange helps to restore mitochondrial homeostasis and to counteract metabolic imbalance, as a defensive mechanism [53]. The core machinery proteins controlling mitochondrial fusion are three GTPase dynamin proteins: MFN1, MFN2 and OPA1. MFNs coordinate the tethering between adjacent mitochondria and consequently OMM fusion; they accumulate at contact areas and can form homo-oligomers (MFN1-MFN1; MFN2-MFN2) and hetero-oligomers (MFN1-MFN2) [54] (Figure 1).

MFNs have been described as composed by a cytosolic N-terminal GTPase domain, a heptad repeat (HR1) domain, two transmembrane anchors and a cytosolic C-terminal HR2 motif. The presentation of this model is based on MFNs yeast topology, which also shows how GTP permits MFNs’ dimerization and how the HR2 domain is able to interact with the N-terminal motif to form a helical bundle, necessary to put membranes in contact and to mediate fusion [55,56]. Recently, Mattie S. and co-workers disputed this model by setting a series of experiments taking advantage of in silico predictive models and in vitro biochemical evidence. Working on animal MFN proteins and highlighting the putative differences between the two topologies, in his model he confirmed a single transmembrane domain with the complete control of oligomerization and consequent fusion by the redox status of two adjacent cysteine residues in the HR2 domain, which is localized exclusively within IMS [57].

OPA1 is a large GTPase responsible for the GTP-dependent fusion of the IMM; its protein structure is mainly localized at the IMS, inserted in the IMM with the N-terminal matrix targeting signal and a transmembrane domain [58]. Mammalian OPA1 gene is transcribed in eight different mRNA variants through alternative splicing of exons 4, 4b and 5b, encoding proteins of 924–1014 amino acids, but the exact function of each isoform is not yet known [59]. Long (L)-OPA1 is the highest molecular weight isoform (~100 kDa) linked to the IMM, that, in case of stress, is cleaved to a soluble short (S)-OPA1 protein (~80 kDa) [60]. L-OPA1 interacts with cardiolipin, a mitochondrial lipid localized in the IMM, with a heterotypic action inducing IMM fusion which is positively regulated by S-OPA1 [61]. In addition to this function, OPA1 acts in the preservation of mitochondrial genome integrity supporting mtDNA replication and distribution [62]; it controls mitochondrial respiratory efficiency and cristae morphology [63].

These processes of fission and fusion are strictly connected to mitochondrial biogenesis [64], another important QC mechanism aimed to preserve cellular homeostasis through the expansion of the mitochondrial network, especially essential in cardiac cells for the generation of an adequate amount of ATP used in muscle contraction. This multistep process includes mtDNA transcription and translation, mitochondrial import of proteins and lipids of nuclear derivation, in order to build new organelles [65] (Figure 1). Given its complexity, biogenesis is controlled by the activation of multiple transcription factors in response to specific stimuli. The main actors working in mitochondrial doubling are two nuclear respiratory factors (NRF1 and NRF2), the estrogen-related receptors (ERR-α, -β, -γ) and PGC-1α [66]. This last one is considered the master regulator of mitochondrial biogenesis, its activity is controlled by an intricate network of signals of different origin (i.e., oxidative stress, exercise or caloric restriction, environmental stimuli) [67]. PGC-1α binds different nuclear transcriptional factors, such as NRF1 and NRF2, which are involved in the control nuclear genes expression linked to mitochondrial respiratory function [68]. Furthermore, PGC-1α can activate transcription factor A (TFAM), a DNA-binding protein responsible for driving the transcription of the mitochondrial genome and of nuclear-encoded proteins with structural properties [69]. Within the major signaling pathways regulating PGC-1α activity, potent inducers of its transcription are members of the transducer of the regulated cAMP response element-binding protein (CREB) family, which are sensors of intracellular Ca^2+^ and cAMP [70]. In addition, the intracellular AMP/ATP ratio is also important in controlling PGC-1α; when increased, for example, after caloric restriction, it activates AMP-activated kinase (AMPK), inducing the expression of PGC-1α and the increase in mitochondrial biogenesis and fatty acid oxidation rate [71]. AMPK is a metabolic sensor which regulates PGC-1α transcriptional activity also through a direct interaction and phosphorylation, possibly in response to high energy consumption [72] (Figure 1). The regulation of PGC-1α expression is controlled also by Sirtuin 1 (SIRT1), a sensor of redox shifts and a controller of mitochondrial metabolism and inflammation, for example after caloric restriction [73], thanks to post-transcriptional regulation of several targets [74]. Among them, SIRT1 deacetylates PGC-1α leading to its transactivation and inducing the transcriptional activity [75].

A schematic representation of mitochondrial fusion and fission machinery and their crosstalk with biogenesis is as follows. (a) Mitochondrial players are involved in fusion, fission and biogenesis. (b) Mitochondrial fission is orchestrated by a series of molecular events involving OMM receptors (MFF, MiD49, MiD51, FIS1), which help the recruitment of DRP1 to the organelle. DRP1 results to be the main protein effector of mitochondrial fragmentation. (c) Mitochondrial fusion is allowed by the tethering properties of MFNs and the function of OPA1 at the IMM. (d) Mitochondrial biogenesis is at the crosstalk of a nuclear/mitochondrial gene reprogramming involving the upregulation of PGC-1α, NRF1/2, TFAM, their nuclear interactions and fusion proteins.

## 4. Impairments of Mitochondrial Dynamics in Coronary Artery Diseases

The most common group of cardiovascular pathologies are the so-called CAD, often characterized by a reduction of the blood flow towards the cardiac tissue due to the development of atherosclerotic plaques at the coronary level [76]. CAD includes the phenotypic manifestations of stable and unstable angina, nonfatal myocardial infarction (MI) and HF [77]. The detrimental effect caused by these pathologies is multifactorial: primarily triggered by the inflammatory state that worsens the atherosclerotic process, then promoted to an insufficient balance between the demand and the supply of oxygenated blood to the tissue and to the reperfusion injury (RI), whether the disease is treated at clinical level or not [15,78,79]. Although major mitochondrial alterations in this context have been described (i.e., intense oxidative stress, increased mitochondrial Ca^2+^ overload, deficiency in ATP production, mitochondrial swelling and cell death [15]), the impairment of QC mechanisms of mitochondria is poorly monitored, but it plays an additional crucial role in CAD and contributes by itself to all those dysregulations (Table 1).

### 4.1. Ischemic Diseases

Overall, fission, fusion and biogenesis are deeply altered after MI, with a significant activation for the first mechanism and the pathological disruption for the last two, as sides of the same coin.

**Figure 2:**
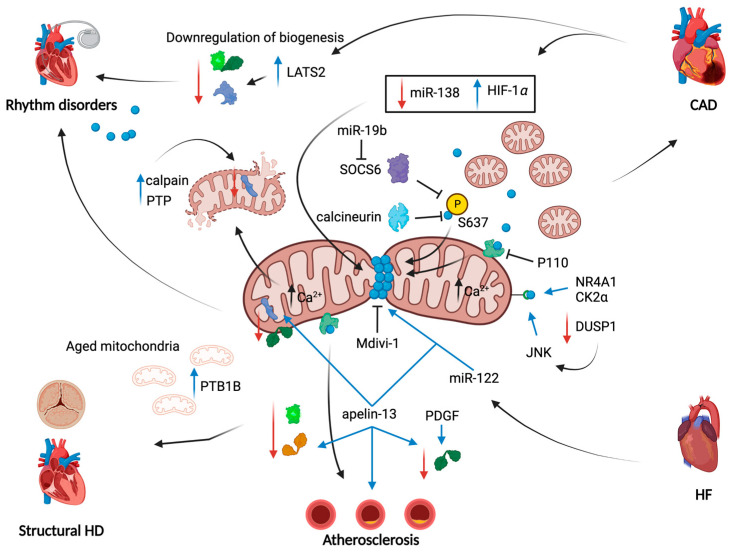
Mitochondrial dynamics alterations in heart diseases. Schematic overview of molecular pathways through which mitochondrial fission, fusion and biogenesis contribute to pathological aspects of heart diseases. Overall, mitochondrial fragmentation is the main common feature of heart diseases reported in this review. The ways by which mitochondria become fragmented are multiple and are aimed to anchor DRP1 to mitochondria. In CAD, a link with the HIF-1α has been found and involves the downregulation of miR-138; a second miRNA, miR-19b, blocks SOCS6 kinase function to prevent DRP1 phosphorylation on Ser-637. The same effect is obtained by the increase in activity of calcineurin. A concomitant decrease in mitochondrial fusion has been reported: in atherosclerosis development, apelin-13 promotes the downregulation of MFNs and OPA1. Also, mitochondrial biogenesis is downregulated through the loss of PGC-1α, NRF1/2 and TFAM expression, especially in CAD and in rhythm disorders in a Hippo pathway-dependent manner. Red arrows: downregulation of a given protein/pathway; blue arrows: upregulation of a given protein/pathway; black arrows: cause-effect link.

Being the main contributor of mitochondrial fission, DRP1 was the most studied protein in this field. After cardiac stress (i.e., prolonged ischemia, inflammation, mechanical stretch), many authors reported an excessive activation of DRP1 and the consequent mitochondrial fragmentation [80,81,82]. Molecular routes by which DRP1 function is overactivated in ischemia and reperfusion (I/R) are multiple (Figure 2). In 2014, Sharp W. and co-workers reported a significant calcineurin-dependent dephosphorylation of DRP1 at S637, which determined its massive mitochondrial translocation [80]. Another mediator impacting on S637 phosphorylation grade is the suppressor of cytokine signaling 6 (SOCS6); it is usually upregulated during I/R and responsible for the inhibition of S637 phosphorylation and the increased DRP1 translocation to mitochondria with consequent fragmentation [81]. Moreover, it has been shown how DRP1 activity may be indirectly dependent on HIF-1α expression, that is upregulated by the decreased expression of miR-138 during I/R [112] (Figure 2). Experiments of reversion valued these molecular intermediates in the control of fission-dependent mitochondrial impairments; indeed, both the repression of SOCS6 by miR-19b injection [81] and the lowered expression of DRP1 induced by miR-138 administration [112], as well as the direct inhibition of DRP1 through mitochondrial division inhibitor 1 (Mdivi-1) [82,94], successfully controlled mitochondrial fragmentation upon cardiac stress.

In some cases, such as the use of Mdivi-1, it was realized that the chronic inhibition of DRP1 cannot be used for therapeutic purposes because it can acquire additional pleiotropic effects in cardiomyocytes. This is the case of the proteolytical cleavage of L-OPA1 and changes in OXPHOS complex proteins’ expression, leading to increased superoxide generation, with impairments of mitochondrial respiration and inhibition of the autophagic process [113]. These results thus suggested that chronic Mdivi-1 treatment could negatively control cardiac tissue function, limiting its potential use in therapy for CAD.

The detrimental effect of enhanced DRP1 expression on cardiac tissue was confirmed also thanks to the use of an additional selective inhibitor of the fission machinery, P110, which acts on the interaction of FIS1/DRP1 fission proteins by inhibiting DRP1 mitochondrial association in several models of I/R. Prevention of excessive mitochondrial fragmentation and the improvement of mitochondrial oxygen consumption, as well as some long-term effects monitored in rats subjected to acute MI, revealed its therapeutic potential [83].

Whatever the regulatory route, the phenotypic effects of the increased DRP1 function result to be major contributors of ROS production, Ca^2+^ overload, cell death and left ventricular (LV) dysfunction. Direct and indirect inhibition of DRP1 mostly reduces MI size, ROS levels in mouse models subjected to coronary artery occlusion and reperfusion damage [80,82]. In a study carried out on CAD patients and investigating the involvement of rs28362491 polymorphism on the NFKB1 gene in the progression of the pathology, its role in the mitochondrial fission process has been discovered. This gene, linked to CAD, encodes for the nuclear factor kappa B (NF-κB) which has been demonstrated to induce, when mutated, increased sensitivity to oxidative stress-induced apoptosis in endothelial cells. NFKB1 gene mutation significantly increased the expression of DRP1 after stimulation with apoptotic stimuli, causing uncontrolled mitochondrial fission and mitochondrial dysfunction [114].

Despite the evidence furnished on the harmful role of DRP1 upregulation in the ischemic disease the cardiac-specific knockdown of DRP1 in mice has also been classified as lethal. Indeed, several mitochondrial impairments, with an excessive state of elongated mitochondria, caused a significant reduction of the mitophagic process that finally led to LV dysfunction and mice death [84]. These data are not considered to be opposed to the previous ones, but further confirm the crucial role of DRP1 (and fission) and its balance in heart homeostasis during life and disease.

Other proteins involved in mitochondrial fission processes deserve attention in CAD. This is the case of DRP1 receptors at OMM, such as MFF, which help the recruitment of the protein at the mitochondrial level. MFF is demonstrated to be activated during I/R, after a cascade involving the nuclear receptor subfamily 4 group A member 1 (NR4A1) and serine/threonine kinase casein kinase2 α (CK2α), which directly act on MFF. A second molecular axis activates MFF, that involves the I/R-mediated downregulation of Dual-specificity protein phosphatase1 (DUSP1) and the activation of c-Jun N-terminal kinase (JNK) leading to the contribution of the RI by apoptosis [115].

This evidence was deepened thanks to MFF deletion studies carried out both in vitro and in vivo: MFF loss reduced exacerbation of mitochondrial fission in I/R, preserving mitochondrial functionality and tissue rescue from damage. It has been discovered that MFF activation leads also to the dissociation of hexokinase 2 (HK2) from mitochondria, which leads to uncontrolled ROS production, PTP opening, cytochrome c release and cellular apoptosis [85].

If on one hand mitochondrial fragmentation depends on the excessive function of fission proteins, on the other hand it can be easily predicted that also MFN1/2 and OPA1 deregulation may be involved in I/R. However, their exact behavior in these kinds of diseases is poorly understood and results in being controversial. Indeed, most studies established to this aim evaluated the genetic ablation impact of the proteins in mouse or rat model diseases. Although this kind of approach helps to elucidate their role once totally deprived, it is not useful to understand their trend in expression and function during disease.

Among the in vivo experiments that were set up to understand the therapeutic potential of improving mitochondrial fusion, a mitochondrial fusion promoter-M1 has been used on rats subjected to I/R, showing a high grade of MI size reduction and associated mortality when administered before ischemia, and a lower but significant effect at the time of reperfusion [86]. This treatment has gained importance for both the selective way of action in counteracting the decrease of MFN2 and OPA1 (fission processes were not affected) and for the reduction of ROS levels, mitochondrial membrane depolarization and swelling with a specific effect on mitochondrial dynamics [86]. With the same purpose, but using conditional cardiac-specific mouse models of YME1L and OMA1 deletion, research studies have addressed the essential role of the L-OPA1 isoform in preserving cardiac function [88]. YME1L and OMA1 are two mitochondrial proteases involved in OPA1 cleavage from L-OPA1 to S-OPA1 forms; in the absence of YME1L, cardiomyocytes experienced enhanced OPA1 proteolysis with greater mitochondrial fragmentation. This macroscopic feature is linked to increased cell death and a heart phenotype resembling that of dilated cardiomyopathy with metabolic drift from lipid to glucose metabolism [88]. Ex vivo experiments on rat hearts subjected to I/R revealed an impaired balance between L-OPA1 and S-OPA1 forms, due to an increase in OMA1 activation and L-OPA1 cleavage that in turn is associated with cardiac dysfunction [116]. Similar findings were obtained by in vitro experiments on cardiomyocytes, where S-OPA1 expression augmented after I/R and changed localization from the mitochondria to the cytosol [117]. OPA1 can be considered a cardioprotective target for therapies against HF; indeed, enhancing fatty acid utilization in vivo through a high fat diet, OPA1 overexpression improved cardiac function by reducing mitochondrial fragmentation [91].

Moreover, some reports described the mitochondrial Ca^2+^ uniport (MCU) as upregulated during I/R, with a consequent increase in mitochondrial Ca^2+^ levels and PTP opening. The consequent calpain expression increase downregulated OPA1-mediated mitochondrial fusion. The final result also in this case is an overactivation of fission, fusion and mitophagy inhibition accompanied by the activation of the apoptotic pathway [87].

Mitochondrial QC mechanisms’ involvement in CAD is not only limited to unbalanced mitochondrial dynamics; in fact, there is evidence that mitochondrial biogenesis is altered, too [118] (Figure 2).

Major findings have been obtained from ex vivo studies, in which human samples of failing hearts have been processed for DNA microarrays. In detail, PGC-1α, the key regulatory partner ERR-α and a series of downstream gene targets have been found downregulated [119]. To this, a significant decrease in mtDNA content and expression of mtDNA-encoded proteins [120], the downregulation of NRF1, TFAM and DNA Polymerase Gamma 1 (POLG1) and decreased expression of proteins involved in mtDNA maintenance are observed [118]. In vivo animal models have confirmed this complex pattern, highlighting the decrease in nuclear-encoded mitochondrial gene expression and in PGC-1α, NRF-2α and mtTFA (a nucleus-encoded protein involved in transcription and replication of mtDNA) gene expression [121]. In three pioneer studies, Fabregat-Andres et al. investigated the peripheral blood concentration of PGC-1α (mRNA and protein) in ST-segment elevation acute myocardial infarction (STEMI) patients at admission time. PGC-1α resting levels detected at hospital admission resulted inversely correlated with the extent of tissue damage in patients with MI [122]. However, PGC-1α expression was increased in blood cells at 72 h and this induction was positively correlated with the infarct size and cardiac remodeling [123], so the authors finally suggested that increase of PGC-1α expression can be part of the recovery response of the mitochondrial protection system to MI, and could be an index of cardiac recovery [124].

A recent work by Chen et al. revealed the possibility of the intersection of the Hippo pathway [125] with mitochondrial biogenesis by detecting an increased expression of large tumor suppressor kinase 2 (LATS2) during I/R in cardiomyocytes. When deleted, it supports cardiomyocyte survival and mitochondrial biogenesis; indeed, it induced the increase in the expression of PGC-1α, in association with the rise of mRNA levels of TFAM and NRF1 attenuating RI [126]. Very recently, an innovative approach aimed to reduce RI has been proposed with much interest in the scientific world: mitochondria transplantation. This technique consists of the use of functionally intact autologous mitochondria from healthy cells or tissue that are directly transplanted in the ischemic tissue right before the reperfusion process. The procedure has revealed incredible results in reducing myocardial damage induced by mitochondrial impairments, restoring sufficient energy for cardiac homeostasis [127]. The mechanism of internalization of the autologous mitochondria in a different tissue has been studied and a key role of NRF2 was revealed. This transcription factor controls mitochondrial biogenesis by influencing the expression levels of other critical transcription factors, such as NRF1 and PGC-1α, as well as by improving purine nucleotide biosynthesis [128]. It has been demonstrated that, during mitochondrial transplantation, both NRF2 expression and its downstream targets’ expression are increased and the cardiac injury is reduced due to the stabilization of the mitochondrial fission and fusion process, reduction of apoptosis and improved mitophagy [129]. Modulation of mitochondrial biogenesis might be an efficient technique to restore cellular homeostasis in cardiac disease and pave the way for promising mitochondrial-specific therapies.

### 4.2. Heart Failure

The last maladaptive stage of most pathologies, not only referring to the cardiac district, is HF. This clinical picture is characterized by several molecular and macroscopic derangements that lead to the absence of an efficient contraction of the cardiac muscle. The first association of mitochondrial fusion and fission proteins in HF dates back to the 2000s, when some groups reported a significant downregulation of OPA1 and MFN2 proteins and FIS1 upregulation as responsible for mitochondrial impairments and apoptosis [89,90]. Of note, the levels of these proteins can vary significantly among the process leading to end-stages of cardiac remodeling, and this fluctuating trend has not been well investigated. In HF, clinicians found the microRNA-122 (miR-122) to be one of the most elevated miRNAs in affected patients [130]. By overexpressing a cardiac-specific version of miR-122, it has been found that increased apoptosis is linked to an increase in DRP1 expression and mitochondrial fission, which contributes to HF [92] (Figure 2). Likewise, analysis on tissue samples from the left ventricular wall of failing canine and human hearts showed an increase in FIS1 and DRP1 proteins and a concomitant reduction of MFN2 and OPA1 expression when compared to control donors [131]. More evidence that matches mitochondrial fission and the onset of HF is given by the establishment of the MFF-KO mouse model. Indeed, MFF-deficient mice died at 13 weeks developing HF, with a cardiac tissue characterized by low mitochondrial density, reduced mitochondrial respiration and augmented mitophagy [132].

A recent study focused on the differences in mitochondrial morphology, dynamics and biogenesis in the two large classes of patients suffering from HF: those with preserved ejection fraction (HFpEF) and those with reduced ejection fraction (HFrEF). HFpEF patients presented mitochondrial fragmentation without changes in protein expression of the fission machinery. HFrEF patients instead showed higher marked mitochondrial structural and morphological changes, with a significant increase in expression of DRP1 and decreased PGC-1α, without differences in the expression of OPA1 and MFN2 [93]. Additionally, in this disease context, PGC-1α played a cardioprotective function in response to cardiac stress like that derived from transverse aortic constriction; in fact, when genetically repressed in mice heart, it leads to an acceleration of the cardiac dysfunction with the final result of HF [133]. A similar role for PGC-1β in controlling cardiac function during HF has been found thanks to the use of PGC-1β-deficient mice, which exhibited an augmented propensity to develop this pathology, due to higher levels of oxidative stress, mitochondrial dysfunctions and a reduction of expression levels of OXPHOS genes [134].

## 5. Impairments of Mitochondrial Dynamics in Heart Rhythm Disorders

Irregular heartbeat characterizes some cardiac pathologies named as arrhythmias, which make up a large percentage of heart diseases. In arrhythmia, the electrical impulse sequences are inconsistent, either too fast, too slow or non-uniform. The improper beating of the cardiac muscle causes an inefficient pump of blood throughout the other vital organs, which may be ultimately compromised [135]. It has been widely recognized that mitochondrial dysfunctions are prominent in the incidence of cardiac rhythm disorders, mainly due to impaired function of intracellular ion channels, to the insufficient energy generation and excessive ROS production, thus leading to an incorrect action potential propagation and adulterated electrophysiology of cardiac myocytes [8,135,136].

### Atrial Fibrillation

Among cardiac arrhythmogenesis disorders, atrial fibrillation (AF) is the most prominent, with an age-related increase in its prevalence [137]. In the last decade, the pathophysiology of AF has been deeply investigated; however, the implications of mitochondrial dysfunctions, in particular the ones which correlate with mitochondrial dynamics, are not fully understood (Table 1).

The first evidence on the involvement of mitochondrial alterations in AF dates back to an early work by Thidemann and Ferrans, in which they found a variation in size (smaller organelles) and number of mitochondria in atrial tissues from patients affected by AF [138]. This finding has been confirmed more recently by Wiersma et al., who assessed that AF is characterized by a shift from tubular mitochondria to a fragmented network, and that this phenotype is associated with cytosolic and mitochondrial Ca^2+^ overload with compromised bioenergetics [139].

Dong et al. used a rabbit model of rapid atrial pacing to demonstrate for the first time that also mitochondrial biogenesis is impaired and is at the basis of AF etiopathology [140]. In his paper, they found that mtDNA levels were reduced in atrial tissue, and the expression of mitochondrial biogenesis transcription factors (i.e., PGC-1α, NRF-1 and TFAM) were decreased together to the mitochondrial respiratory chain complexes. These data correlate with the fact that AF is also characterized by energy metabolism remodeling.

It is important to mention that several risk factors promote AF; however, type 2 diabetes mellitus (T2DM) is the most prevalent [141]. In the study by Shao and co-workers, the T2DM animal group exhibited higher incidence of AF; this event was correlated to a significant reduction in protein expression of PGC-1α, NRF-1 and TFAM, as well as the concomitant downregulation of DRP1, MFN1 and OPA1 [95]. These data suggest impairment of both mitochondrial biogenesis and the fusion-fission process in cardiac tissue. Similar results were obtained by another independent group with the use of a rabbit model of T2DM: their data confirmed a correlation between T2DM and a higher incidence of AF, which is associated with downregulation of mitochondrial biogenesis and fusion and the upregulation of fission-related proteins [142]. Accordingly, Montaigne and co-workers confirmed the presence of fragmented mitochondria directly in patients, as a consequence of MFN1 downregulation in the atrial tissue [96]. Interestingly, patients affected by post-operative AF also exhibited an impaired level of PGC-1α [143], suggesting that mitochondrial biogenesis could be a target platform for AF treatments.

Cardiac arrhythmogenesis involves not only atria but can also occur at the ventricular level. Ventricular fibrillation (VF) is the main cause of sudden cardiac death and is one of the major causes of mortality worldwide [144]. New evidence shows the involvement of proteins which regulate mitochondrial dynamics in VF. Ventricular tissues from rabbit models displayed increased levels of DRP1 and no variation in MFNs [145]; a different result was obtained in an in vivo model of catecholaminergic polymorphic ventricular tachycardia (CPVT) aggravated by the mutation in the trans-2, 3-enoyl-CoA reductase-like (TECRL) gene which encodes for an endoplasmic reticulum protein, published to be associated to inherited arrhythmia [146,147]. The authors showed how TECRL mutation or deficiency triggers significant mitochondrial impairments with decreased mitochondrial biogenesis and fusion protein MFN2 [147]. Interestingly, both AF and VF have been recently seen to be associated with degenerative cardiac PGC-1β dysfunction. It has been demonstrated that PGC-1β null mice exhibit impaired action potential conduction, together with the presence of abnormal mitochondria and altered metabolism [148,149,150].

## 6. Impairments of Mitochondrial Dynamics in Structural Heart Diseases

Structural heart diseases include all those categories of cardiovascular disorders in which an abnormal structure of the heart muscle (valves, walls and chambers) is associated with a pathological function. Any problem with one of the four heart valves can lead to obstructive blood flow. It is very common among the population and it includes a heterogeneous group of valvular impairments with different incidence; they can be congenital [151,152] or develop with age [153,154]. Sometimes, they are associated with other heart diseases, and if not treated in a timely fashion, they might lead to HF. Otherwise, when the structural problems involve the muscle, we can speak about cardiomyopathies (CM). Their classification has evolved along the years [155]. Hypertrophic cardiomyopathy (HCM) [156,157] and dilated cardiomyopathy (DCM) [158,159] are the most prevalent worldwide.

### 6.1. Valvular Heart Disease

In the last decade, mitochondrial dysfunctions have been much more investigated in valvular diseases. In 2017, Rogers and colleagues demonstrated for the first time that calcific regions of human carotid arteries and calcified human aortic valves (CAVS) express very high levels of DRP1 [97]. Microscopic analysis investigated mitochondrial morphology and reported increased mitochondrial fragmentation. This altered morphology was independent from DRP1, as Mdivi-1-mediated inhibition fully restored morphology [97]. Moreover, inhibition of DRP1 reduced the calcification process both in primary human smooth muscle cells and valve interstitial cells via the increase in SRY-box transcription factor 9 expression, suggesting the role of DRP1 in the promotion of osteogenic differentiation [97]. Four years later, in the paper by Morciano et al., an additional puzzle was put down regarding the investigation of several mitochondrial QC mechanisms [98]. Besides the contribution of mitophagy, mitochondrial biogenesis has been evaluated in human calcific aortic valves, showing a significant downregulation of PGC-1α which correlated with aged mitochondria and insufficient mitochondrial turnover [98,160].

In 2022, it was demonstrated that a protein located at the endoplasmic reticulum (ER), the protein tyrosine phosphatase 1B (PTP1B), is expressed in aortic valve stenosis. Immunohistochemistry of a human sample of CAVS displayed increased expression of PTP1B compared with healthy valves. The pharmacological inhibition and the downregulation of PTP1B limited osteogenic differentiation of interstitial valvular cells through the regulation of OPA1 homeostasis [161] (Figure 2). Moreover, drug-induced PTP1B inhibition promoted mitochondrial biogenesis [161], which was demonstrated from our group to be downregulated in CAVS [98].

Bicuspid aortic valve (BAV) is one the most common congenital heart diseases, characterized by the presence of only two cups, which affect almost 1.4% of the population [162]. It has been reported that there is a strong association between BAV and thoracic aortic aneurism (TAA) [162]. A recent study highlighted unbalanced mitochondrial dynamics and mitochondrial bioenergetics in BAV-TAA tissues accompanied with reduced expression of NOTCH1 [99]. The authors used an innovative model, aorta smooth muscle-on-a-chip, to assess the correlation between NOTCH1 downregulation and mitochondrial dynamics. They demonstrated that MFN1 and MFN2 expression was reduced in the BAV-TAA group; on the contrary, DRP1 and MFF levels were increased even if not correlating significantly with the downregulation of NOTCH1 [99]. Although this study has many limitations, it provides the basis for future deeper investigation of the connection between BAV and mitochondrial dynamics dysfunctions, which is still very lacking.

### 6.2. Cardiomyopathies

There are multiple works that demonstrate mitochondrial dynamic impairments in the pathogenesis of DCM [158] (Table 1). A notable example derives from the studies by Ashrafian H., who identified a mouse model, called “Python mouse”, with a missense mutation in the middle domain of the Dnm1l gene that spontaneously developed DCM in a fully penetrant way [163]. C452F mutation in that gene confers to mitochondria an unexpected elongated morphology with the disruption of the fission process; additionally, energetic defects have been found, resembling the DCM phenotype. The chronic elongated mitochondrial phenotype has deleterious consequences, including loss of mitophagy induction, depolarization and impaired ATP production with a progressive decline in OXPHOS [100].

Overall, literature teaches us that almost all animal models characterized by heart-specific KO of a given mitochondrial dynamics-related protein show profound alterations, that all together concur with age to DCM onset. DRP1 KO mice exhibited hypertrophy, LV dysfunction [84] and lethal DCM [101]; when conditionally turned off, they also displayed a decrease in both mitochondrial fission (MFF and FIS1) and fusion proteins (MFN1/2 and L-OPA1), confirming an impaired balance in the mitochondrial dynamics machinery of these hearts [101]. To this regard, Wai et al. demonstrated that cardiac-specific deletion of YME1L triggers the onset of DCM through the activation of OMA1 and induced proteolysis of OPA1 [88], subsequently showing fragmented mitochondria in cardiomyocytes isolated from these mice hearts. These data were confirmed by an independent study, in which heterozygote OPA^+/−^ mice had a concomitantly slow development of DCM and exhibited a reduction in the mitochondrial fusion process [164]. Additionally, the deletion of MFF, as investigated by Chen et al., triggered the death of mice at 13 weeks, due to severe DCM [132]. Interestingly, the same study showed that the simultaneous deletion of MFF and MFN1 prevented cardiac defects, probably balancing mitochondrial fission and fusion mechanisms. However, with the cardiac-specific deletion of both MFN1 and MFN2, DCM is lethal to mice, with a severe mitochondrial fragmentation, reduced mtDNA content and alterations in the biogenesis process [38,102,103]. Recently, these results have started to be partially confirmed also in humans, who are affected by DCM and low levels of MFN1 and mitochondrial fragmentation in cardiomyocytes [165]. In the same diseased patients, but from a study of an independent research group, mitochondrial biogenesis was found as a compensatory mechanism, extremely upregulated, sustained by the upregulation of PGC-1α, NRF1/2 and TFAM [166]. Abnormal biogenesis in DCM human samples correlated with morphological and respiratory dysfunctions [167].

## 7. Insights on the Role of Mitochondrial Dynamics in Atherosclerosis

Atherosclerosis is a multifactorial degenerative disease characterized by alterations of the artery wall, which loses elasticity due to the accumulation of Ca^2+^, cholesterol, inflammatory cells and fibrotic material.

Compelling evidence highlighted a key role for mitochondrial dynamics in endothelial dysfunction, a hallmark of early atherosclerosis (Table 1). Atherosclerosis is an increasing complication in patients with diabetes; therefore, many studies have focused on the role of endothelial dysfunction in diabetes-induced CVD. Accordingly, it was found that mitochondrial fragmentations and FIS1 expression were increased in venous endothelial cells isolated from patients with diabetes mellitus [104]. A similar trend was also observed in cultured human aortic endothelial cells (HAEC), which showed a loss of the mitochondrial network and an increase in FIS1 and DRP1 expression upon glucose [104] or adipokine RBP4 [168] treatment. This phenotype was rescued by the inhibition of mitochondrial fission [104]. Moreover, DRP1 inhibition by Metformin was remarked to improve the endothelial activity and to prevent the atherosclerotic lesions in apolipoprotein E-deficient mice [105]. Metformin does not directly act on DRP1, but it triggers AMPK activation that in turn reduces the levels of the fission protein; additionally, AMPK promotes DRP1 Ser-637 phosphorylation to limit its translocation to mitochondria. Of note, only the AMPK-α2 isoform blocks the effect of Metformin on mitochondrial dynamics when depleted. All these studies confirm that alterations of mitochondrial fission contribute to the development of atherosclerosis in diabetes.

Chehaitly A. and co-workers recently investigated the role of fusion in endothelial cells’ responses to flow since shear stress-mediated dilation is another risk factor for atherosclerosis. They found that the disturbed flow decreased OPA1 expression and mitochondrial length in vitro. In addition, shear stress-mediated dilation was reduced in the arteries of OPA1^+/−^ mice. The absence of OPA1 did not influence the acetylcholine-mediated dilation, thus OPA1 deficiency may selectively affect the endothelial response to flow [106]. Beside the endothelial component, vascular smooth muscle cells (VSMC) also play a central role in the formation of atherosclerotic plaque. Abnormal proliferation and migration of VSMCs promotes plaque formation and they are closely related to plaque vulnerability. The activation of VSMC by platelet-derived growth factor (PDGF) was found to induce a loss of the mitochondrial network associated with a significant decrease in MFN2 levels, without affecting the number of mitochondria. Inhibition of mitochondrial fragmentation avoided the hyperproliferation response of VSMCs to PDGF, demonstrating that the conversion of VSMCs in the proliferating phenotype was associated with mitochondrial morphology [108] (Figure 2). Along this line, another study observed that MFN2 overexpression significantly reduced VSMCs’ proliferation and intima thickening in vivo by suppressing AKT-mediated survival pathways [109,110]. These results are consistent with studies demonstrating that mitochondrial fission also plays a role in VSMCs’ hyperproliferation. Interestingly, apelin-13, a stimulator of VSMCs’ proliferative activity, reduced not only the expression of MFN2, MFN1 and OPA1, but it also increased the expression levels of DRP1 in human aortic cells [111]. The same group found that apelin 13 accelerated atherosclerosis in the aorta, while the pharmacological inhibition of DRP1 restored the physiological mechanism of fission and attenuated the proliferating activity of VSMCs in APOE^−/−^ mice [111]. Marsboom et al. also confirmed that the inhibition of DRP1 by Mdivi-1 restored the mitochondrial network and slowed the proliferation of pulmonary artery smooth muscle cells [107].

Arterial calcification is another hallmark of atherosclerosis and DRP1 seems to play an important role in this phenotype. In fact, DRP1 promotes human cardiovascular calcification via regulating osteogenic differentiation [97]. DRP1 inhibition attenuates matrix mineralization, cytoskeletal rearrangement, leading to the lowering of VSMC calcification. Taken together, these findings strongly suggest that both the enhancement of fission and the reduction of fusion have a relation with atherosclerosis pathophysiology.

Among the mitochondrial quality control mechanisms, the role of mitochondrial biogenesis in the onset of atherosclerosis has drawn the attention of the scientific community. The overexpression of PGC-1α in VSMCs significantly ameliorated HCD-induced atherosclerotic plaque formation in rabbit models. High levels of PGC-1α in VSMCs inhibited the switch of VSMCs in the proliferating status [169]. In contrast, Steins’ paper reported that the mouse model PGC-1α^−/−^/APOE^−/−^ exhibited a reduction of total body and visceral fat weight, but it did not show differences in the atherosclerotic burden compared to PGC1α^+/+^/APOE^−/−^. The decreased inflammation as a consequence of reduced visceral fat in PGC-1α KO animals could explain why they did not develop atherosclerosis [170]. Moreover, it was reported that PGC-1α polymorphisms correlated to the atherosclerosis onset and its complications [171,172,173]. Although the role of mitochondrial biogenesis in atherosclerosis is not well established, collectively, these data make it clear that modulation of mitochondrial dynamics could be a possible therapeutic strategy to slow atherosclerosis progression and plaque formation.

A schematic overview of molecular pathways through which mitochondrial fission, fusion and biogenesis contribute to pathological aspects of heart diseases is as follows. Overall, mitochondrial fragmentation is the main common feature of heart diseases reported in this review. The ways by which mitochondria become fragmented are multiple and are aimed to anchor DRP1 to mitochondria. In CAD, a link with the HIF-1α has been found and involves the downregulation of miR-138; a second miRNA, miR-19b, blocks SOCS6 kinase function to prevent DRP1 phosphorylation on Ser-637. The same effect is obtained by the increase in the activity of calcineurin. A concomitant decrease in mitochondrial fusion has been reported: in atherosclerosis development, apelin-13 promotes the downregulation of MFNs and OPA1. Additionally, mitochondrial biogenesis is downregulated through the loss of PGC-1α, NRF1/2 and TFAM expression, especially in CAD and in rhythm disorders in a Hippo pathway-dependent manner. Red arrows: downregulation of a given protein/pathway; blue arrows: upregulation of a given protein/pathway; and black arrows: cause-effect link.

## 8. Still Little Data from Clinical Studies

From data reported so far, the accumulation of fragmented and damaged mitochondria results to be closely related to the development of CVD. Despite the therapeutic potential of fission/fusion modulation in CVD, there are currently few active clinical trials. Much preclinical evidence supports the potential effectiveness of molecules targeting mitochondrial dynamics and biogenesis to treat CVD. Future challenges will be to turn these molecules into treatments, but clinical studies are still needed to validate their efficacy and safety.

In 2013, the clinical trial of Metformin in patients with peripheral artery disease (PAD) was started to evaluate its beneficial effects on skeletal muscles (NTC01901224). Unfortunately, the results of this clinical study have not been published because it was terminated prematurely due to lack of funds. Based on preliminary data showing that DRP1 inhibition reduces atherosclerotic plaque formation in ApoE KO models [105], another trial aimed to investigate the role of DRP1 in human macrophages and to identify new therapeutic targets has been established (NCT03980548). The study is currently ongoing. A non-randomized interventional study that began this year aims to compare mitochondrial dynamics between patients operated on for ascending aortic aneurysm, type A aortic dissection and a control group (NCT05434481).

Many clinical trials have investigated sodium-dependent glucose transporter 2 (SGLT2) inhibitors in treating diabetic-induced cardiac diseases. Particularly, a phase III double-blind randomized study (NCT01131676) evaluated the effects of Empagliflozin, as a SGLT2 inhibitor, in patients with T2DM and high cardiovascular risk. The inhibitor’s administration reduced cardiovascular mortality and hospitalization [174]. Experimental studies unveiled the positive effects of SGLT2 inhibitors on mitochondrial biogenesis by PGC-1α and SIRT1 activation [175]. SGLT2 inhibition reduces FIS1 and enhances expression levels of MFN2 and OPA1 both in vitro and in vivo [176]. Although the effects on mitochondrial dynamics in humans are still to be confirmed, SGLT2 represents a promising target against cardiac disease. Melatonin was also found to be a cardioprotective molecule against different cardiovascular pathologies. Preclinical studies recently investigated the role of melatonin on vascular calcification and demonstrated that melatonin promoted OPA1-mediated mitochondrial fusion and decreased VSMCs’ calcification [177,178]. A meta-analysis of seven randomized clinical trials revealed that melatonin administration attenuated LV ejection fraction and heart dysfunctions [179]. The effects of melatonin on mitochondrial dynamics have not yet been evaluated in humans, and further trials are needed to confirm the data obtained in the experimental studies. Taking all of this into consideration, the lack of clinical studies has not yet allowed for the validation of the effectiveness of preclinical strategies in improving mitochondrial morphology and metabolism in cardiovascular disease.

## 9. Mitophagy and Mitochondrial Unfolded Protein Response

Mitophagy and mitochondrial unfolded protein response (mtUPR) are considered additional mechanisms of QC, because they are aimed to restore a condition of mitochondrial integrity following stress. The first one is expected to remove damaged organelles through a localized and selective process of lysosomal digestion [180], the second one is a cellular protective response which follows the accumulation of unfolded proteins in the mitochondrial matrix [181].

Balanced mitophagy counteracts several mitochondria-mediated pathological processes, especially those linked to mtROS overproduction and the activation of the chronic inflammatory response. An obvious example is represented by atherosclerosis, in which the activation of the canonical pathway attenuates NLRP3 inflammasome activity, IL-1β maturation and mtROS levels; this helps to stabilize atherosclerotic plaques and to downregulate the inflammatory response [11,182]. Mitophagy is also induced after I/R events with cardioprotective properties which allow cellular ATP preservation [183] and cell survival [184]. These notions make us understand how essential this process is. Inefficient mitophagy has been found to be linked to HF [185,186], vascular calcification [98] and congenital heart disease [187]; here, the targeting of mitophagy has improved the disease traits.

Otherwise, the causes and the effects of the activation of mtUPR in heart diseases are still little known; nevertheless, the induction of mtUPR is considered to be a pro-survival pathway [188] to the same extent as mitophagy. Some in vitro stressors (i.e., complex I inhibition, Hsp90 loss of function, unfolded proteins) and in vivo changes in hemodynamic parameters (i.e., pressure overload in HF) trigger the transient activation of mtUPR in the heart. Through a linked nuclear reprogramming, which is induced by mtUPR and largely independent from ATF5, an upregulation of an adaptive gene response is able to restore mitochondrial dysfunctions and cardiac contractility [189,190].

## 10. Conclusions

From all these notions, in a controversial appearance, we can exclude the possibility of clearly defining a disease trait as characterized by a well-defined pattern of proteins’ expression and function related to mitochondrial dynamics and biogenesis impairments. Although mitochondrial fragmentation and disruption of mitochondrial biogenesis are recognized as predominant mechanisms in heart diseases, the protein pool involved in these routes is not equally described among reports. As a consequence, selective therapeutic approaches against CVDs with the use of inhibitors of mitochondrial fission or promoters of mitochondrial fusion and biogenesis are still in their infancy. One thing stands out to be clear and homogeneous among the studies analyzed: the need for balance among all QC mechanisms, including mitochondrial dynamics, mitophagy and biogenesis, at each stage of heart development. This is a delicate balance that is difficult to measure and to be targeted, but once dysregulated, leads to pathological effects.

## Figures and Tables

**Table 1:** Representative list summarizing alterations in mitochondrial dynamics in different cardiovascular disorders.

Cardiovascular Diseases	Protein Expression Alterations	Phenotype Manifestations	Ref.
Ischemia-reperfusion injury	DRP1 excessive activations	Increase of mitochondrial fragmentation ROS production, Ca^2+^ overload, cell death and LV impairment	[80,81,82,83]
	Persistent DRP1 downregulation	Excessively elongated mitochondria, significant downregulation of the mitophagic process, LV dysfunction and cell death	
	MFF loss	Reduction of mitochondrial fission, microvascular mitochondrial structure amelioration	[84]
	MFF activation	Opening of PTP, apoptosis and infarct size expansion	[85]
	MFN2 and OPA1 reduction	Mitochondrial membrane depolarization and swelling	[86]
	OPA1 downregulation	Overactivation of mitochondrial fission and fusion, inhibition of mitophagy and apoptosis	[87]
Heart Failure	OPA1 proteolysis upregulation	Enhanced mitochondrial fragmentation, increased cell death and metabolism alteration	[88]
	OPA1, MFN2 downregulation and FIS1 upregulation	Mitochondrial impairment and apoptosis	[89,90]
	OPA1 upregulation	Improved cardiac function, reduced mitochondrial fragmentation	[91]
	DRP1 upregulation	Increase of mitochondrial fragmentation Increase mitochondrial fission and increase apoptosis Higher mitochondrial structural morphological modifications	[92,93]
Coronary artery disease	OPA1 overexpression	Reduction of MI size	[91]
	DRP1 inhibition	Preservation of mitochondrial network and morphology, reduction of MI size	[80,81,82]
	DRP1 inhibition/OPA1 proteolytic cleavage	Uncontrolled mitochondrial fission and mitochondrial alterations	[82]
	DRP1 upregulation	Reduction mitochondrial fission, mitochondrial dysfunction and apoptosis	[94]
Atrial Fibrillation	Reduction of DRP1, MFN1 and OPA1	Alteration in mitochondrial fission-fusion and mitochondrial biogenesis	[95]
	MFN2 downregulation	Mitochondrial fragmentation	[96]
Calcific aortic valve	DRP1 upregulation	High mitochondrial fragmentation	[97,98]
	DRP1 inhibition	Reduction of the calcification process	
Bicuspid aortic valve/thoracic aortic aneurysm	DRP1 and MFF reduction/MFN1 and MFN2 downregulation	Unbalanced mitochondrial dynamics	[99]
Cardiomyopathy	DRP1 Disruption	Elongated mitochondrial morphology and disruption in mitochondrial fission process, mitophagy alteration	[100,101]
	MFN1/2 deletion	Severe mitochondrial fragmentation and reduced mtDNA	[102,103]
Atherosclerosis	Upregulation of FIS1 expression	High mitochondrial fragmentation	[104,105]
	Induction of DRP1 and FIS1 expression	Loss mitochondrial network	[97]
	DRP1 inhibition	Lower VSM calcification, matrix mineralization Reduction of mitochondrial degradation and prevent atherosclerosis lesions Restoration of mitochondria network and slow pulmonary artery smooth muscle proliferation cells	[106]
	OPA1 downregulation	Reduction of mitochondrial length	[107]
	MFN2 downregulation	Loss of mitochondrial network	[108]
	MFN2 overexpression	Reduction of VSMCs proliferation	[109,110]
	MFN1/2, OPA1 downregulation and DRP1 upregulation	Mitophagy impairment Accelerate atherosclerosis	[111]

## Data Availability

Not applicable.
